# Remembering Professor S. M. Channabasvanna

**DOI:** 10.4103/0019-5545.64574

**Published:** 2010

**Authors:** Prof. S. K. Chaturvedi

**Affiliations:** Professor and Head, Department of Psychiatry, NIMHANS, Bangalore, India. E-mail: skchatur@gmail.com

**Figure:**
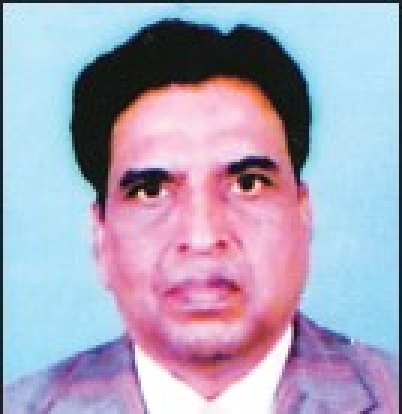
25^th^ April 1932 – 23^rd^ April 2010

Professor S. M. Channabasvanna (Sindagi Marallusidappa Channabsavanna) was born in Northern Karnataka in April 1932. He would have been 78 years old on the 25th of April, a couple of days after he passed away!

He did his schooling in Tiptur and then joined MBBS at Mysore Medical College and subsequently did MD in General Medicine which he passed out with a Gold Medal. Professor SMC (as he was known by all of us) joined DPM at AIIMH in 1956. After qualifying as a psychiatrist, he worked initially at the Dharwad Mental hospital for a short period, following which he joined AIIMH. He was first an Associate Professor and in 1977 became a Professor of Psychiatry. In addition, he was the Deputy Medical Superintendent for nearly 8 years and then became the Medical Superintendent in 1979. He took over as the Head of the Department of Psychiatry at NIMHANS in the year 1982. He was also the Dean at NIMHANS.

He became the Director of NIMHANS in 1989 and continued as the Director between 1989 and 1997 and was instrumental in NIMHANS becoming a Deemed University in 1994. He became the first Vice Chancellor/Director of NIMHANS. He was the first Emeritus Professor of Psychiatry of NIMHANS.

Prof. SMC was one of the giants of Indian psychiatry and, of course, psychiatry in Karnataka. He was President of the Indian Psychiatric Society. He was the Honorary Editor of the Indian Journal of Psychiatry from 1985 to 1988 and the President of the Indian Association of Social Psychiatry.

Dr. SMC won many awards and accolades and continued to be a member of many distinguished boards and committees. Even after retirement, he continued to be a Government nominated advisor to the psychiatric centers at Tezpur, Ranchi, and IHBAS in Delhi. He was a pioneer in many areas of Psychiatry in India.

He started the Family Psychiatry services and Family ward, and Forensic psychiatry services at NIMHANS. He was one of the key members involved in the formulation of the Mental Health Act. He also spearheaded several new initiatives in the mental health scenario in the country including Improvement of Mental hospitals and the current NMHP. He was also instrumental in starting the first collaborative initiative with the National Human Rights Commission for the rights of the mentally ill.

He was a mentor and teacher to many of the psychiatrists who are professors, directors and heads of departments today. Sense of calmness, ability to solve problems, excellent people skills, sense of humor and approachability were his hallmark qualities.

He was very well known for his practical and common-sense approach to difficult problems, particularly in the area of forensic issues. He would give very sensible advice which one could not find in any text book. Such advice was often sought by faculty, students, fellow professionals and even the legal professionals! Everyone was at ease with him. The juniormost student could enter his office without any trepidation and go back with a smile. His humility was another endearing quality. He would eat in the canteen with everyone else and when on rounds would often taste the food which was being distributed to patients, to ensure quality.

After retirement, he continued to be active in academics and would be seen sitting in the front row of all academic programs taking notes seriously. Such was his dedication and commitment to the field of mental health. The psychiatry fraternity of India will remember him for his warmth, sensible advice and constant zeal and energy that he brought to the field of psychiatry, and he will be missed a lot.

